# Renal Surgery

**Published:** 1895-01-19

**Authors:** 


### PROGRESS IN SURGERY.

### RENAL SURGERY.

**Suppurative Pyelo-nephritis.**—Keyes contributes[1] a paper on the surgical aspect of nephritis, and concludes that (1) healthy urine is sterile; (2) purulent urine is always microbic; (3) microbic infection takes place from within the body by a number of methods in the course of disease, and is often brought about by instrumental manœuvres on the part of the surgeon; (4) a healthy organism and vigorous bladder may cope successfully with microbic invasion, and rid itself spontaneously, or with a little aid, of all damage arising therefrom, showing little or even no inflammatory response; (5) a suitable condition of the patient is essential to the propagation and perpetuation of inflammatory phenomena upon the urinary tract, after microbic invasion; (6) this condition, intensified by traumatism and physical weakness, is most intense when there is vesical distension with atony, and when the ureters are dilated and the kidneys involved in the changes incident to tension below—namely, atrophy and sclerosis above, with or without surface catarrh; (7) under these circumstances, surgical pyelo-nephritis is most likely to declare itself as a result of microbic infection from below (occasionally from above), in the course of suppurative disease or after operative interference; (8) asepsis, antisepsis, and sterilisation of urine are ends to be aimed at in genito-urinary surgery. Much can be done by local means in a prophylactic and curative way; little by internal medication; and possibly as much, more than by any other means, by flushing the urinary passages with natural mineral waters.

It was found[2] that nitrate of silver was by far the best agent for overcoming the effects of local contamination, while corrosive sublimate was better where the tissues were involved. R. F. Weir reports[3] a case in which he performed nephrectomy for "surgical kidney" following gonorrhœa, and another[4] in which the same affection followed scarlet fever. Both cases recovered. The question arises as to how often this affection is limited to one kidney, so as to make it amenable to surgical treatment, and Weir quoting[5] various statistics concludes that 20 per cent. of cases are capable of being attacked surgically.

**Moveable Kidney** is a term which has given rise to some confusion, and Macdonald Brown, therefore, proposes to classify them as follows[6]: (1) Fixed Displacements.—Where the mobility is always less than one inch in any direction. (2) Moveable Kidney.—Where the mobility exceeds the last figure, and the organ is mobile in its relaxed capsule, or between the muscular wall of the abdomen and the peritoneum, and has no meso-nephron. (3) Floating Kidney.—Where there is a meso-nephron and the organ floats free in peritoneal cavity.

For this condition Harvey Reed thinks[7] that palliative treatment rarely succeeds, and that the only safe and successful treatment of a moveable kidney is by a radical operation by which the kidney is replaced, and firmly anchored by a number of sutures. If this is not done there is always danger of serious organic disease of the kidney, which may sooner or later prove fatal, and if not the patient becomes a nervous suffering invalid. On the other hand, P. Segond considers[8] that in nephroptosis it is only when the pain is unbearable that the medical treatment is to be dispensed with and nephropexy resorted to. Weber[9] had a case of pseudo-membranous colitis, which supervened in a woman, coinciding with floating kidney complicated by hydronephrosis, and which was cured by nephropexy. Dugardin-Beaumetz[10] explains this by the fact that pseudo-membranous enteritis always occurs in neurotic individuals, and says it is not to be wondered at that a laparotomy, or some other operation, should exert a powerful influence on the nervous state of such patients. Verhoogen and Godard[11] point out that moveable kidney may occur in nulliparous women and young girls, and therefore maintain that there is a complete independence between enteroptosis and moveable kidney. Such cases are to be attributed to the abuse of corsets. From the point of view of symptoms they divide the patients into three classes: (1) The neurotics—very impressionable, with painful points in the back, &c.; (2) the dyspeptics—this is the most numerous class; (3) patients troubled with uterine symptoms brought on by the floating kidney.

**Hydronephrosis** is caused, according to Bland Sutton,[12] by an incomplete obstruction to the flow of urine, or, if complete, intermittent, because sudden complete and permanent obstruction tends to cause atrophy rather than dilatation of the kidney. Most surgeons agree[13] that in the presence of a hydronephrosis, *a fortiori*, if suppurating, the best course is to remove it by nephrectomy, provided the other organ is in good working order. As it is essential to ascertain the condition of the other kidney, it is advised to open the abdomen for exploratory purposes, and then, after closing the abdomen, to remove the kidney affected, if necessary, through a lumbar incision.

**Renal Calculus.**—W. Dendy and W. Eagles report[14] a case of oxalic acid calculus treated by lumbar nephrolithotomy. The patient, however, did not submit himself to operation until the kidney had become so disorganised that its removal was necessary four months later. During this interval of time abscesses in and about the kidney occurred, and the man had an attack of pyæmia. He subsequently recovered. Demons and Poussons have collected eighteen cases (including three of their own) of calculus anuria which were subjected to operation with a mortality of 33⅓ per cent. This compares very favourably with those treated expectantly, for Leguen gives the mortality of the latter as 71·5 per cent. The authors think that calculos anuria indicates operation as urgently as does intestinal obstruction. Access to the renal pelvis is to be obtained by an incision through the convexity of the kidney. This operation being so simple and free from danger is to be preferred to ureterotomy or pyelotomy, unless the location of the calculus has been accurately determined. Duret[16] has formed the following conclusions with regard to the treatment of branching calculi of the kidney: (1) When suppuration of the pelvis of the kidney does not exist, or when tke lesions are not very extensive, the extraction of a branching calculus may be accomplished by simple nephrotomy; but this operation should be preceded by crushing the stone, that is to say, by nephrolithotrity. (2) If the branching stone is large and associated with considerable pyonephrosis of old standing, it is usually impossible to resort to nephrectomy at once. The operation must then be performed in successive stages: (*a*) Evacuation of the purulent collection by nephrotomy on the convex edge of the gland. (*b*) Lithotrity of the stone *in situ*, so as to extract it piecemeal. (*c*) Nephrectomy of the degenerated kidney, and ablation of the sac. Postnikoff records[17] a case in which, on opening the abdomen for a hydronephrotic condition, the ureter was found greatly dilated and thickened, while probing showed that its vesical end was blocked by calculi. The latter—fourteen in number—were extracted, after which the ureter was tied close to the bladder and excised together with the kidney. The patient made a complete recovery. As regards the removal of a whole ureter, the case is believed to be unique.

**Contusions and Lacerations of the Kidney** may occur, according to Morris,[18] without at the time, or till some considerable time afterwards, causing any of the characteristic signs of the injury, viz., hæmaturia, alteration in the amount of urine, or tumour in the renal region. If the damage to the kidney is complicated by injury to the liver, spleen, intestine, bladder, or lungs, or by extensive fractures of the ribs or pelvis, the shock is so profound and the hæmorrhage so extensive, that surgical aid is of no avail, and a fatal termination inevitably follows in the course of several hours, or at longest a few days. When, however, the injury is limited chiefly, if not entirely, to the kidney, we must consider (1) whether the immediate treatment shall be palliative or operative, and (2), whether, if an operation is done, the damaged kidney should be removed, or the wound packed with antiseptic gauze and drained, and the kidney allowed to remain. The answer to the first question will depend, not upon the presence or absence of hæmaturia or primary anuria, but upon the presence or absence of a distinct and increasing swelling in the loin. Hæmaturia is not a reliable symptom, and only requires a lumbar exploratory incision (*a*) if from its amount or continuance the strength of the patient is being exhausted; (*b*) when it causes an accumulation of blood clots in the bladder. The rule should be to cut down upon the kidney in the loin, and unless hæmorrhage can be controlled, the kidney should be removed. Nor is primary anuria an indication for operative treatment, because temporary suppression of urine is a common result of shock. If, however, it continues for more than twenty-four or thirty-six hours, and after the other symptoms of shock have passed off, or if it supervenes some days after the accident, the lumbar exploration ought to be made, as an outlet for the urine is urgently required. The presence of a distinct and increasing swelling in the renal region makes an early incision through the loin imperative; it is the only way to cut short the progress of the case; to limit the extravasation into the peritoneal tissues, to check or prevent suppuration, to prevent sloughing of the peritoneum, and to save the life of the patient. No hard and fast rule can be laid down as to the question of nephrectomy. If the kidney is completely pulped; if hæmorrhage cannot be controlled except by ligaturing the vessels at the hilum; if the surface of the kidney is extensively contused and lacerated; if hæmorrhage seems likely to recur in a patient whose life has been already endangered by loss of blood; or if suppuration and septic infection are threatened, nephrectomy should be performed. In cases where the damage is not so extensive it is justifiable and safe to perform a partial nephrectomy; but in cases in which the pelvis of the kidney only is torn; or in which the extravasated fluid is chiefly or entirely urine, not blood; as well as those in which the parenchyma is superficially wounded, the bleeding stopped, and the kidney and the wound in an apparently healthy state, nephrectomy ought not to be performed.

[1] Am. J. Med. Sc., June, 1894, p. 613. [2] Op. Cit. and N. Y. Med. J., June 16, 1894, p. 756. [3] Med. Rec. N. Y., July 21, 1894, p. 82. [4] Med. Rec. N. Y., Sept. 15, 1894, p. 325. [5] Op. Cit. [6] Quoted by Med. Chron., Sept., 1894, p. 474. [7] J. Am. Med. Ass., May 12, 1894, and Int. Med. Mag., June, 1894, p. 392. [8] Med. Week, July 20, 1894. [9] Med. Week, Nov. 2, 1894. [10] *Ibidem*. [11] Gaz. de Gynécol, March 1, 1894. [12] Quoted in Med. Rec., Aug. 4, 1894, p. 138. [13] *Ibidem*. [14] Lancet, Sept. 29, 1894. [15] Ann. des Malad. des Org. Génito-urin., 1894, No. 2, and Am. J. Med. Sc., Aug., 1894. [16] Med. Week, Oct. 5, 1894, p. 488. [17] Vratch. No. 12, 1894, and Brit. Med. J., July 14, 1894. [18] Clin. J., Aug. 1, 1894, p. 217.

